# Supplementary material for: An Interpretative Phenomenological Analysis of the lived experience of sensuality expression among women over 50 years of age in Nigeria
Source: PLoS One. 2023 Jun 1;18(6):e0285362. doi: 10.1371/journal.pone.0285362 (PMC10234539; doi:10.1371/journal.pone.0285362)
Supplement: S1 File — (DOCX) [file pone.0285362.s001.docx]

**TRANSCRIPT FOR SENSUALITY EXPRESSION**

**TRANSCRIPT OF PARTICIPANT 1**

**INTERVIEWER:** Good Morning Madam. I am happy that you have consented to participate in this research, and I appreciate your commitment. The first section is meant to help us know each other. Please tell me your name, age, marital status, and your highest level of education.

**PARTICIPANT 1:** I am 53 years old, and married. My highest level of education is a First degree.

**INTERVIEWER:** Thank you. Please note that I will not use your real name in the report, but a coded form. Now, the next section contains questions dealing with the main research. Please let me know if you find it uncomfortable answering any of the questions, so that we may determine how to proceed.

The first area we are going to talk about is about the physical changes have you experienced in your body since you turned 50 years? “How do these changes make you feel”. “How have you expressed these changes to others, including your partner, male colleagues and younger females?” “How do you perceive yourself since you turned 50?”” Do you feel attractive to others?” “Are you attracted to other men?” “What are you doing to remain attractive and cover up your ageing looks?

**INTERVIEWER:** Since you turned 50, have you noticed any change in your body?

**PARTICIPANT 1:** Hmmm, the first changes that I noticed was in my breast. This left breast, that time I complained to Mrs. A (she mentioned a name). It seems as if it was becoming larger, bigger, and painful. This is something which I have not been experiencing before.

**INTERVIEWER:** And what have you done about it?

**PARTICIPANT 1:** I came to the hospital, they treated me then

**INTERVIEWER:** I am sorry to hear about that, but how about now?

**PARTICIPANT 1:** There is still pain, but not as much as before

**INTERVIEWER:** Did you go back to the hospital to complain about the pain, did they tell you what it was?

**PAR TICIPANT 1:** Yes, they tested me. He referred me for a test in Ibadan

**INTERVIEWER:** MECURE? (A popular diagnostic center)

**PARTICIPANT 1:** Yes, MECURE. I went there for Mammography. When I got the result or report, they said there was nothing wrong.

**PARTICIPANT 1:** That is the main change that was conspicuous. Apart from that, my breasts are no longer firm, they sag and droop like an old woman’s

**INTERVIEWER:**  How does that make you feel?

**PARTICIPANT 1:** I do not like the way my breasts dropdown. Though I am not fat like most women my age with big breasts, but my sagging breasts spoil the show. I think it somehow reduces my attractiveness. I wish my breasts would remain pointed and firm, like before.

**INTERVIEWER: What are you doing about it?**

**PARTICIPANT 1:** To look more attractive like I used to be, I try to support my sagging breasts with a padded bra to make it look bigger and upright. This will bring out my beauty.

**INTERVIEWER:** Thank you for sharing that with me. Has there been any other changes in your body, for instance in your body weight or looks?

**PARTICIPANT 1:** No

**INTERVIEWER:** Your size remained the same?

**PARTICIPANT 1:** Yes, no change

**INTERVIEWER:** Is there any other change?

**PARTICIPANT 1:** I stopped menstruating 4 years ago. But it doesn’t disturb me like many people do say, when they reach the stage of menopause, that they feel somehow. But in my own case, I feel nothing

**INTERVIEWER:** Nothing? Please explain what you mean by saying it does not disturb you like many people. How does it disturb other people?

**PARTICIPANT 1:** I hear some people complain of feeling hot and sweating excessively. some say they have changes in their moods. But in my case, nothing, nothing whatsoever. It doesn’t disturb me, it doesn’t affect me

**INTERVIEWER:** Since you turned 50, how do you relate with other men? Do you find them attractive?

**PARTICIPANT 1:** When I was younger, even in marriage, there were a few instances when I found other men physically attractive. I sometimes fantasized about having romantic relationships with them. It was fun then, but now, I feel it is unheard of, for a woman of my age to feel attracted to men or engage in any form of romance. If you have such feelings, you must kill it immediately I would say that I still relate well with men. I am polite to my male colleagues. Our interactions are purely professional. I do not encourage any social interactions with other men, especially fellow teachers. And I no longer find them attractive.

**TRANSCRIPT OF PARTICIPANT 2**

**INTERVIEWER:** Good Morning Madam. I am happy that you have consented to participate in this research, and I appreciate your commitment. The first section is meant to help us know each other. Please tell me your name, age, marital status, and your highest level of education.

**PARTICIPANT 2:** I am 56 years old, and married. My highest level of education is a First degree.

**INTERVIEWER:** Thank you. Please note that I will not use your real name in the report, but a coded form. Now, the next section contains questions dealing with the main research. Please let me know if you find it uncomfortable answering any of the questions, so that we may determine how to proceed.

The first area we are going to talk about is about the physical changes have you experienced in your body since you turned 50 years? “How do these changes make you feel”. “How have you expressed these changes to others, including your partner, male colleagues and younger females?”” “How do you perceive yourself since you turned 50?”” Do you feel attractive to others?” “Are you attracted to other men?” “What are you doing to remain attractive and cover up your ageing looks?

**INTERVIEWER:** I am, going to start by asking if you have noticed any changes in your body since you turned 50?

**PARTICIPANT 2:** The one that is so typical of my age group

**INTERVIEWER:** Which one is that?

**PARTICIPANT 2:** The menopause

**INTERVIEWER:** Ok, when did you observe this change?

**PARTICIPANT 2:** 6 years ago

**INTERVIEWER:** What are the changes your saw because of menopause?

**PARTICIPANT 2:**  Just the usual common experiences which are common to many people, like profuse sweating and the likes. But the most troublesome was the feeling of heat. It sometimes comes on so bad and unexpectedly that I sometimes feel like fainting. Thank God it didn’t last for too long.

**INTERVIEWER:** What did you do about it?

**PARTICIPANT 2:** I didn’t do anything about it because I know it is normal and it is to be expected. I guess because one is also relieved that the monthly women palaver is finally over

**INTERVIEWER:** How are you now?

**PARTICIPANT 2:** The signs have almost completely disappeared now

**INTERVIEWER:** Any other change? In your size or your looks? Are you gaining weight, do you think you look different?

**PARTICIPANT 2:** There is no change in my weight, my weight has remained stable, I am lucky, there is no struggle for weight control

**INTERVIEWER:** Any other change?

**PARTICIPANT 2:** My hairline has receded, and my hair is now fluffy and sparse.

**INTERVIEWER:** Are you comfortable with that?

**PARTICIPANT 2:** That is not a problem, I now own a variety of wigs: long and short; curly or wavy which I wear in different styles to project the image of someone with a full head of hair. They are classy and elegant.

**INTERVIEWER:** Have you noticed any other changes?

**PARTICIPANT 2: Yes.** The appearance of wrinkles on my face and neck I have noticed some wrinkles on my face and neck, it is a reminder that I am no longer young, that I am growing old.**”**.

**INTERVIEWER:** Are you comfortable with that?

**PARTICIPANT 2:** Well, I know it’s a necessary part of growing old. We all wish to grow old, yet have a beautiful body that attracts men, don’t we all? I know I do. (laughs)

**INTERVIEWER:** Can you explain what you mean by that statement?

**PARTICIPANT 2:** I am not as attractive as I was before I turned 50. I feel I am getting old. I don’t look as beautiful as I was when I was younger. My skin is no longer as spotless as before, maybe because I no longer use the expensive body cream as before. There was a time I was using a particular cream to lighten the dark skin of my face and to smoothen the wrinkles on my face and neck to maintain my youthful look. Everyone I know including my family, remarked how toned my skin was, and I was happy they noticed. I used to really pay attention to my body then to be attractive to men, not necessarily my husband, but men generally. But now I stopped using that expensive cream. I have come to accept the change, and this is who I am now, they must accept me for who I am

INTERVIEWER: How have you reacted to these changes?

**PARTICIPANT 2:** I try not to let the changes in my looks bother me. I used to be very romantic when I was younger, I even had other men in my life. So, I stayed in shape to keep their interests. But when I turned 50, it became difficult to keep up with their expectations. I accepted the fact that men no longer find me attractive because it became obvious that they preferred younger women to me. So, I stopped dating other men before they sack me. But it was fun while it lasted though.

**TRANSCRIPT OF PARTICIPANT 3**

**INTERVIEWER:** Good Morning Madam. I am happy that you have consented to participate in this research, and I appreciate your commitment. The first section is meant to help us know each other. Please tell me your name, age, marital status, and your highest level of education.

**PARTICIPANT 3:** I am 52 years old, and married. My highest level of education is a First degree.

**INTERVIEWER:** Thank you. Please note that I will not use your real name in the report, but a coded form. Now, the next section contains questions dealing with the main research. Please let me know if you find it uncomfortable answering any of the questions, so that we may determine how to proceed.

The first area we are going to talk about is about the physical changes have you experienced in your body since you turned 50 years? “How do these changes make you feel”. “How have you expressed these changes to others, including your partner, male colleagues and younger females?” “How do you perceive yourself since you turned 50?”” Do you feel attractive to others?” “Are you attracted to other men?” “What are you doing to remain attractive and cover up your ageing looks?

**INTERVIEWER:** The first question is on changes in your body. Since you turned 50, have you observed any change in your body?

**PARTICIPANT 3:** I feel tired easily, I am not as physically strong as when I was younger. I don’t have the stamina to work for longer periods as before. I also discovered that my tummy is becoming bigger, and I am gaining weight generally. I am no longer the slim, elegant lady that I was. I also discovered that I have started having some wrinkles on my neck. I think my face is also darker than before. I look old somehow (laughs). I wish I could have my trim figure back and remain attractive like I was in my younger days to be appreciated by others

**INTERVIEWER: How do you feel about the changes?**

**PARTICIPANT 3:** I am not comfortable with these changes in my body, especially the wrinkles. I mean, how can I have wrinkles at this age? I still wish I would always remain attractive as I was when I was younger.

**INTERVIEWER:** What are you doing about the wrinkles?

**PARTICIPANT 3:** To achieve a youthful look, I use wrinkle-repair creams and fill-ins to mask the wrinkles. Though I think it is part of life, part of growing old

**INTERVIEWER:** Does these changes affect your relationship with your colleagues, especially the male ones?

**PARTICIPANT 3:** No, not really, but some of them jokingly remark that I am getting old, that you’re an old woman now. Then I ask them “are you not old yourself”. Don’t mind them.

**INTERVIEWER:** HOW do you feel about their remarks

**PARTICIPANT 3:** Most times I do not let their comments bother me, but sometimes I wish I could have my old body back

**INTERVIEWER:** Are you seriously doing anything to have your old body back?

**PARTICIPANT 3:** Not really, I have accepted those changes as inevitable part of growing old. I don’t expect I will always look attractive. I cannot compete with the younger ladies anyway.

**INTERVIEWER:** Any other change since you turned 50?

**PARTICIPANT 3:** I cannot remember any other

**INTERVIEWER:** What is your experience with menopause?

**PARTICIPANT 3:** Oh, that is something else.

**INTERVIEWER:** How do you mean, please explain

**PARTICIPANT 3:** My experience started 6 years ago when I was 46 years. I was experiencing intermittent ceasation of menses. That was a trying period because I felt it has come too soon. Also, I was in dilemma whether to remove my intra-uterine contraceptive device or not. But a health worker advised that I leave it for a while until I was sure the menses has stopped. This was followed by severe hot flushes and sweating that sometimes become embarrassing.

**INTERVIEWER:** How so?

**PARTICIPANT 3:** While others feel moderate heat, mine will be severe. I will break out in beads of perspiration, my clothe will even become wet. It often happens suddenly that people nearby will ask if I was okay or not. Then it will subside just as suddenly as it appeared.

**INTERVIEWER:** Are you attracted to other men?

**PARTICIPANT 3:** You know women will always be attractive to men, I believe that. Men will always find women attractive. Many of them will express their feeling. They will tell you that you are beautiful, things like that. There are so many of them, but I am not attracted to any of them.

**TRANSCRIPT OF PARTICIPANT 4**

**INTERVIEWER:** Good Morning Madam. I am happy that you have consented to participate in this research, and I appreciate your commitment. The first section is meant to help us know each other. Please tell me your name, age, marital status, and your highest level of education.

**PARTICIPANT 4:** I am 53 years old, and married. My highest level of education is a First degree.

**INTERVIEWER:** Thank you. Please note that I will not use your real name in the report, but a coded form. Now, the next section contains questions dealing with the main research. Please let me know if you find it uncomfortable answering any of the questions, so that we may determine how to proceed.

The first area we are going to talk about is about the physical changes have you experienced in your body since you turned 50 years? “How do these changes make you feel”. “How have you expressed these changes to others, including your partner, male colleagues and younger females?” “How do you perceive yourself since you turned 50?”” Do you feel attractive to others?” “Are you attracted to other men?” “What are you doing to remain attractive and cover up your ageing looks?

The first question I’m going to ask is about changes in your body. Since you turned fifty, have you experienced any change in your body

**PARTICIPANT 4:** When I look in the mirror, I see that I am looking older, maybe when I’m powdering my face, I can see a difference in how I used to look in my younger days and now

**INTERVIEWER:** What are the differences?

**PARTICIPANT 4:** Though there are no wrinkles in my face and my colour is not changing, but I can see that the face is not smooth as before, and it is ageing. Other than that, my weight is not changing much

**INTERVIEWER:** These change in your look; how do you feel about it?

**PARTICIPANT 4:** That makes me feel somehow because I still wish to remain young and beautiful. But now, I feel my youthful look is gradually going

**INTERVIEWER:** Any other changes?

**PARTICIPANT 4:** Hmm, towards the end of last year, I started experiencing menopause. That was at the age of 52

**INTERVIEWER:** How does these changes make you feel?

**PARTICIPANT 4:** There is no difference in the way I feel, I don’t feel anything. I only feel relief because I’m free from the hassles of monthly bleeding. Some of my friends often complain of many bizarre things happening to them during menopause, but as for me, nothing happened to me, apart from the cessation of my menses. I have no complaints, thank God.

**I****NTERVIEWER:** Did you experience any other changes?

**PARTICIPANT 4:** Yes, I remember now. At some time after I turned fifty, I was feeling pains in the bones and joints in my legs, it became difficult to rise from sleeping or sitting positions. I got my relief after I joined a health fitness club for exercises. Now, I am as fit as a fiddle

**INTERVIEWER:** What about your husband, how is the change in your look affecting him or your relationship?

**PARTICIPANT 4:** It doesn’t affect our relationship. He has no complaints

**INTERVIEWER:** What about your male colleagues?

**PARTICIPANT 4:** Is it not 2 months ago, I travelled to Ibadan, I met an old classmate of mine when I was in secondary school, he just exclaimed, ‘’haa, this woman you have become old now, you’re not as fine as before’’

**INTERVIEWER:** Did that matter to you?

**PARTICIPANT 4:** No, not at all, I know its age that is affecting me. I don’t expect to remain forever young

**TRANSCRIPT OF PARTICIPANT 5**

**INTERVIEWER:** Good Morning Madam. I am happy that you have consented to participate in this research, and I appreciate your commitment. The first section is meant to help us know each other. Please tell me your name, age, marital status, and your highest level of education.

**PARTICIPANT 5:** I am 50 years old, and divorced. My highest level of education is a First degree

**INTERVIEWER:** Thank you. Please note that I will not use your real name in the report, but a coded form. Now, the next section contains questions dealing with the main research. Please let me know if you find it uncomfortable answering any of the questions, so that we may determine how to proceed.

The first area we are going to talk about is about the physical changes have you experienced in your body since you turned 50 years? “How do these changes make you feel”. “How have you expressed these changes to others, including your partner, male colleagues and younger females?” “How do you perceive yourself since you turned 50?”” Do you feel attractive to others?” “Are you attracted to other men?” “What are you doing to remain attractive and cover up your ageing looks?

**INTERVIEWER:** Since you turned 50, have you experienced any changes in your body

**PARTICIPANT 5:** Emm no, but sometimes, maybe while sleeping, I always feel bone pains, that my bone will be paining me in the night, I don’t want to stand up or I feel reluctant to stand up. I always have bone whatever; I don’t know how to put it

**INTERVIEWER:** And you think that is because you are 50 and over

**PARTICIPANT 5:** Yes, I think so because it has not been affecting me before I turned 50

**INTERVIEWER:** What have you done about the bone pains?

**PARTICIPANT 5:** I used pain killers, but sometimes ago, when it got bad, I went to the hospital. I was given a stronger Analgesic and I was given some drugs to make my bones stronger, like Calcium and Iron tablets

**INTERVIEWER:** Did that work?

**PARTICIPANT 5:** Yes, the pain subsided gradually with time

**INTERVIEWER:** What other changes have you observed?

**PARTICIPANT 5:** Hmm, you know I have not been menstruating again

**INTERVIEWER:** Yes, that’s a change

**PARTICIPANT 5:** Actually, the menstruation stopped since when I was 47. At that time, I was having hot flashes, sweating and some memory problems. I started forgetting things

**INTERVIEWER:** And since you turned 50, are there further changes regarding menstruation

**PARTICIPANT 5:** Not really, the complaints persisted for a while. But in the last few months or so, I have little or no complaints

**INTERVIEWER:** Any itching or vaginal discharges

**PARTICIPANT 5:** No, no no no

**INTERVIEWER:** What about your look, or your size, any changes?

**PARTICIPANT 5:** Ha, actually when people see me, they don’t really believe that I’m up to that age. They said that is how I’ve been looking since I’ve been a youth. Because there was a time someone called me and said he Mrs. (mentions her name), you have not really changed your stature. So, I have not changed really. Thank God for that. (Laughs)

**INTERVIEWER:** So, are you comfortable with your looks?

**PARTICIPANT 5:** I don’t know o, I don’t know o. You know its people that look at you and most times you don’t see yourself, its people that know what you really look like. But I love the complements I get from people regarding my look. I would say that I love the way I look

**TRANSCRIPT OF PARTICIPANT 6**

**INTERVIEWER:** Good Morning Madam. I am happy that you have consented to participate in this research, and I appreciate your commitment. The first section is meant to help us know each other. Please tell me your name, age, marital status, and your highest level of education.

**PARTICIPANT 6:** I am 56 years old, and married. My highest level of education is a National Certificate in Education.

**INTERVIEWER:** Thank you. Please note that I will not use your real name in the report, but a coded form. Now, the next section contains questions dealing with the main research. Please let me know if you find it uncomfortable answering any of the questions, so that we may determine how to proceed.

The first area we are going to talk about is about the physical changes have you experienced in your body since you turned 50 years? “How do these changes make you feel”. “How have you expressed these changes to others, including your partner, male colleagues and younger females?” “How do you perceive yourself since you turned 50?”” Do you feel attractive to others?” “Are you attracted to other men?” “What are you doing to remain attractive and cover up your ageing looks?

The first question I want to ask is that since you turned 50, have you experienced any change in your body?

**PARTICIPANT 6:** Yes, Menopause.

**INTERVIEWER:** What was your experience?

**PARTICIPANT 6:** When the thing started, my menstruation will cease for 3 months, at times, I will be feeling that am I pregnant again, later it will appear again and cease for another few months, sometimes disappearing to come back after 9 months, then it will come back. It was on and off like that until it finally disappeared 4 years ago when I was 52 years.

**INTERVIEWER:** Are there any other changes you observed in your body since you turned 50?

**PARTICIPANT 6:** There was a time that I was having lower abdominal pains, like menstrual pains, and some vaginal discharges. This went on for a little while. I went to the hospital to complain. The Doctor said it was most likely an infection in the Pelvis. I was given some Analgesic and Antibiotics which cleared the infection, and the pain subsided. After that I started experiencing pain during sexual intercourse, my libido was low, my vagina would remain dry even during intercourse and I wasn’t looking forward to the experience. I would say I began to dread the experience. It took a toll on our relationship because we both didn’t know what was causing it and we certainly didn’t know what to do about it.

**INTERVIEWER:** How long has that gone on for?

**PARTICIPANT 6:** Since I was 52 years, that was 4 years ago

**INTERVIEWER:** Thank you for sharing these experiences with me. Now, let’s talk about other changes that you have observed in your body since you turned 50. Like physical changes.

**PARTICIPANT 6:** I have gained more weight; my tummy is fat, and I am generally not as pretty as when I was younger. My face is a bit darker than before and recently; I noticed some wrinkles in my face, not much though. But it is the beginning of aging. I observed that old age is setting in gradually

**INTERVIEWER:** How do you feel about these changes in your body?

**PARTICIPANT 6:** I feel my body is getting old. I wish that my skin would glow as it used to when I was younger. There was a time I was using a cream to lighten the skin of my face, everyone I know started to remark that I was toning, some said I was bleaching my skin. Even my husband and children complained about it, so I stopped using it, now my face has remained darker than my body. Also, there is nothing I have not done to reduce my tummy, it just won’t go down. So, when I wear some nice office dresses, it really doesn’t look quite nice, because of the tummy. So, now, I mostly wear loose dresses. I don’t let it bother me anymore. I am okay. I have accepted it as part of growing old

**TRANSCRIPT OF PARTICIPANT 7**

**INTERVIEWER:** Good Morning Madam. I am happy that you have consented to participate in this research, and I appreciate your commitment. The first section is meant to help us know each other. Please tell me your name, age, marital status, and your highest level of education.

**PARTICIPANT 7:** I am 54 years old, and married. My highest level of education is a First degree.

**INTERVIEWER:** Thank you. Please note that I will not use your real name in the report, but a coded form. Now, the next section contains questions dealing with the main research. Please let me know if you find it uncomfortable answering any of the questions, so that we may determine how to proceed.

The first area we are going to talk about is about the physical changes have you experienced in your body since you turned 50 years? “How do these changes make you feel”. “How have you expressed these changes to others, including your partner, male colleagues and younger females?” “How do you perceive yourself since you turned 50?”” Do you feel attractive to others?” “Are you attracted to other men?” “What are you doing to remain attractive and cover up your ageing looks?

**PARTICIPANT 7:** Since I turned fifty, if it is physically, I’ve not experienced much because I always mind my physique, I try to remain trim and not to gain excess weight. But when it comes to…maybe the physiological side there are changes, although I had an operation like five years ago…so since then I’ve been able to enter menopause prematurely so that’s the issue.

**INTERVIEWER: Was that before you were 50?**

**PARTICIPANT 7:** Yes…that was at 49. I had an operation at 49

**INTERVIEWER:** What sort of operation was that

**PARTICIPANT 7:** I had fibroid operation. The fibroid and the whole womb were removed

**INTERVIEWER:** So, how did you experience the menopause? What are the changes that you saw after the menopause?

**PARTICIPANT 7:** Sometimes, I feel depressed somehow. Sometimes, I can’t just explain why I feel what I feel about some things. You know…maybe sometimes in reaction to issues or maybe in response to relationships. I feel so sad at times. Even in my body, I just feel like some things were not the way it was sometimes before the operation. Like my mood for example. I feel my spirit is down most times, very little catches my fancy. I was not interested in many of the things happening around me. That feeling was severe for the first few months after the operation. The Doctor says that it was the sudden or drastic withdrawal of certain hormones because of the removal of my ovaries during the operation that was causing the depressed feelings. The Doctor advised that I will be put on treatment if the feeling persisted or becomes worse. Thankfully, the feelings started getting better. The depressed feeling disappeared about 1 year after the operation.

**INTERVIEWER:** What other changes have you noticed in your body since you turned 50?

**PARTICIPANT 7:** There is not much change in my weight since I turned fifty? The other change that I observed is maybe in diet, I discovered that there was a certain diet that, normally, I so much love or go after. I discovered now, that I don’t have much interest in that particular diet anymore. It’s not that I don’t have appetite for it, but I just don’t crave it like before. I guess I’m being conscious that, most of such diet are not really what one needs currently. I disliked getting fat. I always pay attention to my physical appearance and more importantly, what I consume; I put in effort to remain trim and not to gain excess weight. I am aware that a trim figure preserves my youthful look and gives me satisfaction and a sense of accomplishment.

**INTERVIEWER:** Is there any other change that you can remember, your skin, wrinkles, on any part of your body?

**PARTICIPANT 7:** There are grey hairs

**INTERVIEWER:** How does the appearance of grey hair make you feel?

**PARTICIPANT 7:** I’m happy about it, that at least I’m getting old, I’m no more a kid you know…I’m a matured adult, something like that.

**INTERVIEWER:** Okay.

**PARTICIPANT 7:** Initially, when I started seeing some grey hair, I was plucking them out. Later when they became more, I started to dye my hair black, because I thought it was too early to wear grey hair. So now, my hair is black.

**TRANSCRIPT OF PARTICIPANT 8**

**INTERVIEWER:** Good Morning Madam. I am happy that you have consented to participate in this research, and I appreciate your commitment. The first section is meant to help us know each other. Please tell me your name, age, marital status, and your highest level of education.

**PARTICIPANT 8:** I am 55 years old, and married. My highest level of education is a First degree.

**INTERVIEWER:** Thank you. Please note that I will not use your real name in the report, but a coded form. Now, the next section contains questions dealing with the main research. Please let me know if you find it uncomfortable answering any of the questions, so that we may determine how to proceed.

The first area we are going to talk about is about the physical changes have you experienced in your body since you turned 50 years? “How do these changes make you feel”. “How have you expressed these changes to others, including your partner, male colleagues and younger females?” “How do you perceive yourself since you turned 50?”” Do you feel attractive to others?” “Are you attracted to other men?” “What are you doing to remain attractive and cover up your ageing looks?

**PARTICIPANT 8:** The visible changes that I recognized are in my hair. My hair is turning white and becoming thin

**INTERVIEWER:** How do you feel about that?

**PARTICIPANT 8:** It makes me look older than my age. I plan to start applying hair dye to darken it and return it to the way it looked in my younger days. Then, I used to have dark full hair. We all know that grey hairs portray one as old. I do not want that image yet.

**INTERVIEWER:** What other changes have you noticed?

**PARTICIPANT 8:** My breasts are no longer firm; they are flat and flabby. How I wish that my breasts will remain firm and pointed; instead of sagging the way they do now.

**INTERVIEWER:** Your breasts look firm to me now.

**PARTICIPANT 8:** I try to package my sagging breasts well in a good, pointed bra. (laughs). Like now, my breasts are well-padded in a firm bra, and that makes my dresses fit more and make me look more attractive. It takes some creativity to package them well in a good, pointed bra. How I wish ageing would not have come with all these changes in my body, I wish I can have my old body back.

**INTERVIEWER:** What other changes have you noticed?

**PARTICIPANT 8:** There is the fact that I have grown fat and have become less attractive in the last few years. I admire women my age who still have beautiful shapes. I wish I look like them. I also wish to maintain my pretty figure to remain attractive.

**INTERVIEWER:** What can you attribute to this change?

**PARTICIPANT 8:** I can say now that I am over 50, the children demand less attention, they can take care of themselves to a large extent, so I have more time for myself. Also, my financial situation is better, so I pay more attention to my body, I consciously watched my weight so that I don’t put in excess weight. Most importantly, I have peace of mind. The family is settled, and most of the things that I worried about are settled e.g., completing our own building, paying school fees in higher institutions. My children are doing fine, and I am generally contented with my lot in life

**INTERVIEWER:** What is your experience with menopause?

**PARTICIPANT 8:** I reached menopause at exactly 50 years. In fact, I didn’t know it was menopause that set in. I got very sick, I had problems with my legs, I couldn’t walk, I thought it was arthritis. It was at the hospital that I was told it was menopausal problem. I had joint pains and back pains. I visited the hospital, I was advised to eat more fruits, exercise more and I was given some drugs whose name I can’t remember now. The problem subsided gradually, and it eventually disappeared. Apart from that one, I really did not experience any other problem associated with menopause

**TRANSCRIPT OF PARTICIPANT 9**

**INTERVIEWER:** Good Morning Madam. I am happy that you have consented to participate in this research, and I appreciate your commitment. The first section is meant to help us know each other. Please tell me your name, age, marital status, and your highest level of education.

**PARTICIPANT 9:** I am 54 years old, and married. My highest level of education is a First degree.

**INTERVIEWER:** Thank you. Please note that I will not use your real name in the report, but a coded form. Now, the next section contains questions dealing with the main research. Please let me know if you find it uncomfortable answering any of the questions, so that we may determine how to proceed.

The first area we are going to talk about is about the physical changes have you experienced in your body since you turned 50 years? “How do these changes make you feel”. “How have you expressed these changes to others, including your partner, male colleagues and younger females?” “How do you perceive yourself since you turned 50?”” Do you feel attractive to others?” “Are you attracted to other men?” “What are you doing to remain attractive and cover up your ageing looks?

**PARTICIPANT 9:** The first one, I noticed was that I always have headache. And this headache is constant.

**INTERVIEWER:** Always there?

**PARTICIPANT 9:** Always there. I had to go to the hospital and once they checked me, they told me it’s a sign of menopause.

**INTERVIEWER:** Okay

**PARTICIPANT 9:** Headache with shivering. Like as if I have malaria, but it was going on for too long, so they told me that it’s a sign of getting to the peak of menopause that I should relax, that there’s nothing much to it, then I just took my mind off it. And suddenly, at the peak, my monthly blood went off and then I stopped menstruating again

**INTERVIEWER:** So how long ago was that?

**PARTICIPANT 9:** About four years ago immediately I clocked 50.

**PARTICIPANT 9:** One thing that I’ve noticed is that I’m not as strong as I was before, because we are keeping poultry. Before now, I could take care of the two or three hundred birds that we have but as I’m getting older, I cannot perform those duties very well again. I get easily tired. Same with other house chores. Also, in my place of work, I always get tired most of the time. Before, if they asked me to take lessons for 2 hours straight, I will not hesitate, I’ll just take it…take it up. But now, by the time I finish with the 2 hours’ lesson, I will be so tired, craving to take Coke. So that is what I noticed about my strength and stamina in recent times.

**INTERVIEWER:** Did that bother you?

**PARTICIPANT 9:** Yes, when I realized that this is not good for my health; I started to exercise in form of walking for at least 30 minutes every day. Gradually, I started regaining my stamina.

**TRANSCRIPT OF PARTICIPANT 10**

**INTERVIEWER:** Good Morning Madam. I am happy that you have consented to participate in this research, and I appreciate your commitment. The first section is meant to help us know each other. Please tell me your name, age, marital status, and your highest level of education.

**PARTICIPANT 7:** I am 58 years old, and widowed. My highest level of education is a First degree.

**INTERVIEWER:** Thank you. Please note that I will not use your real name in the report, but a coded form. Now, the next section contains questions dealing with the main research. Please let me know if you find it uncomfortable answering any of the questions, so that we may determine how to proceed.

The first area we are going to talk about is about the physical changes have you experienced in your body since you turned 50 years? “How do these changes make you feel”. “How have you expressed these changes to others, including your partner, male colleagues and younger females?” “How do you perceive yourself since you turned 50?”” Do you feel attractive to others?” “Are you attracted to other men?” “What are you doing to remain attractive and cover up your ageing looks?

**INTERVIEWER:** Are there one or two things that have changed since you were 50 some years back. So, what has changed in your body?

**PARTICIPANT 10:** I don’t have much change at all, but… (Sighs), I don’t have much power like I was when I was younger.

**INTERVIEWER:** That is a change. Your strength is not as much as before. What other changes have you noticed?

**PARTICIPANT 10:** I’ve put on weight.

**INTERVIEWER:** That’s a change! (Both laugh). That’s a change, you have added weight and how do you feel about that?

**PARTICIPANT 10:** Initially when I started gaining weight, I was very fat and unattractive, but I have worked on shedding the excess weight.

**INTERVIEWER:** How did you work on shedding the excess weight?

**PARTICIPANT 10:** I made efforts to correct it. I deliberately reduced my meal size and eat light meals at night. I worked hard to achieve and maintain my ideal weight. I was avoiding junks, eating well, and exercising as well. All these I did to maintain my figure and preserve physique. I stopped eating things that will add too much to my weight, I watch what I eat.

**INTERVIEWER:** Are you satisfied with your weight now?

**PARTICIPANT 10:** Yes. All that hard work finally paid off

**INTERVIEWER:** Okay. Which other change?

**PARTICIPANT 10:** My hair used to be full before. But now, it isn’t as full as before. But I believe I still have some…this is my hair, no attachment and it’s still dark right?

**INTERVIEWER:** Yes, it is still dark. Are you okay with this?

**PARTICIPANT 10:** Yes. I am quite happy with my hair. I can still make my hair into many styles. I am particularly happy because the hair is still dark. It gives me a youthful look coupled with my trim stature. Grey hair somehow adds to your age. (laughs)

**INTERVIEWER:** Okay. What about menopause?

**PARTICIPANT 10:** Menopause for me started early. I think at the age of 40. I stopped menstruating after I gave birth to my last born at the age of 40. So since then, I’m feeling fine.

**INTERVIEWER:** Since your last child at 40, you have not had menstruation?

**PARTICIPANT 10:** No, and I’m cool with it. I don’t have to worry about family planning. God gave me a permanent one free of charge

**INTERVIEWER:** Okay, what are the changes that you saw after menopause?

**PARTICIPANT 10:** I didn’t have any strange thing in my body.

**INTERVIEWER:** Nothing happened? Nothing different from before? Except that the menses stopped. You said menopause came early for you, at 40. Did that bother you?

**PARTICIPANT 10:** Initially I didn’t realize menopause had set in. I had a baby, I normally don’t menstruate when I am breastfeeding, not until after I weaned the baby off breast. That time, I expected my menses back, but it never returned. I didn’t have any symptoms of menopause maybe because I was lactating, and maybe because I was young, and I was maintaining my body.

**INTERVIEWER:** How were you maintaining your body?

**PARTICIPANT 10:** You know I just had a baby. I was breastfeeding, I was gaining weight. So, I deliberately worked to shed all the excess weight and thereafter maintain my weight. I was avoiding junks, eating well, and exercising.

**INTERVIEWER:** So, are you…Is there anything you would like to change in your body back to what it was before? Or you are satisfied?

**PARTICIPANT 10:** I’m satisfied. Even, you know that I don’t have husband now, what else am I going to use all that thing for?

**TRANSCRIPT OF PARTICIPANT 11**

**INTERVIEWER:** Good Morning Madam. I am happy that you have consented to participate in this research, and I appreciate your commitment. The first section is meant to help us know each other. Please tell me your name, age, marital status, and your highest level of education.

**PARTICIPANT 11:** I am 55 years old, and widowed. My highest level of education is a First degree.

**INTERVIEWER:** Thank you. Please note that I will not use your real name in the report, but a coded form. Now, the next section contains questions dealing with the main research. Please let me know if you find it uncomfortable answering any of the questions, so that we may determine how to proceed.

The first area we are going to talk about is about the physical changes have you experienced in your body since you turned 50 years? “How do these changes make you feel”. “How have you expressed these changes to others, including your partner, male colleagues and younger females?” “How do you perceive yourself since you turned 50?”” Do you feel attractive to others?” “Are you attracted to other men?” “What are you doing to remain attractive and cover up your ageing looks?

**PARTICIPANT 11:** One thing I noticed that is bothering me is that I am not as physically strong as before. I am not able to do many things unlike before. In fact, I told my children that it seems as if I am lazy nowadays. I don’t know what is wrong with me. Then they will say mummy you are not a young woman now; you are getting old

Then, I noticed some memory loss (laughs). I forget things easily, sometimes it is embarrassing, when at work for instance, even at meetings, I wanted to talk about two things, before I finish one, I would have forgotten the other thing I want to talk about

**INTERVIEWER:** It is normal to experience some form of forgetfulness as we age. But to slow down the process and improve your memory, you can be involved in things that would give you mental challenges, like playing games. We can join the children in playing games, especially those that involve some calculations which challenges the brain. It is a fact that we are growing older daily, that our body would not remain the same over time, but there are things we can do to help ourselves. Any other changes?

**PARTICIPANT 11:** Well, I think my experience with menopause was not unusual. I experienced menopause at age 48. The menses was going and coming intermittently for about 1 year before it completely stopped. Many of the things I experienced were things that I have either heard or read about. I was sweating more, I experienced hot flashes, I started feeling numbness in my limbs, I think the memory loss started around that time as well

**INTERVIEWER:** How did you feel about those changes?

PARTICIPANT 11: The onset of menopause was a welcome idea, because I was no longer sexually active because of the death of my husband, so already I felt like I wasn’t a woman any longer. So, the cessation of menses just completed the transition. I was told the numbness might be due to Vitamin deficiency, so I started taking Multivitamins, specifically the ones for women over 50. And I think it worked

**INTERVIEWER:** Which other changes do you observe in your body since you turned 50?

**PARTICIPANT 11:** I have gained some weight; people say it is due to menopause. I also see that my fair complexion is not as light as before, especially my face, it is getting darker

**INTERVIEWER:** How did these changes make you feel?

**PARTICIPANT 11:** I think apart from the fact that I am growing older, I would say maybe I stopped paying so much attention to my body. In truth, I am not interested in attracting the attention of men. I do not want to remarry because I need to concentrate on taking care of my young children. So, I would say I’m okay.

**TRANSCRIPT OF PARTICIPANT 12**

**INTERVIEWER:** Good Morning Madam. I am happy that you have consented to participate in this research, and I appreciate your commitment. The first section is meant to help us know each other. Please tell me your name, age, marital status, and your highest level of education.

**PARTICIPANT 12:** I am 50 years old, and married. My highest level of education is a First degree.

**INTERVIEWER:** Thank you. Please note that I will not use your real name in the report, but a coded form. Now, the next section contains questions dealing with the main research. Please let me know if you find it uncomfortable answering any of the questions, so that we may determine how to proceed.

The first area we are going to talk about is about the physical changes have you experienced in your body since you turned 50 years? “How do these changes make you feel”. “How have you expressed these changes to others, including your partner, male colleagues and younger females?” “How do you perceive yourself since you turned 50?”” Do you feel attractive to others?” “Are you attracted to other men?” “What are you doing to remain attractive and cover up your ageing looks?

**INTERVIEWER:** What changes have you experienced in your body since you turned fifty?”

**PARTICIPANT 12:** Changes are normal. Initially when I was younger, before I got married, even after I got married, I was very active, but now it seems the physical strength has reduced. I experience fatigue nowadays, sometimes I feel dizzy at times. I am finding it increasing difficult to maintain my weight, I must pay particular attention to my weight now unlike before. I would not like to lose my shape at this age, so I eat less and exercise more.

I can also say that I am not as pretty as when I was in my 40s for example, my complexion is getting darker. Also, my hair is thinning out, it’s not as full as before, and now I have some grey hairs. Though I could associate the greyness to stress that I passed through during a recent challenge that I faced.

**INTERVIEWER:** How does these changes make you feel?

**PARTICIPANT 12:** Not terrible, but it’s a realization that one is advancing in age, no one remains youthful forever. Though, I think I now need to pay more attention to my skin, my face, and my look generally than I normally would before now, to appear beautiful and attractive. So, now that my children are grown up, I have more time to pay attention to my looks, to remain attractive and appear youthful still. I also realize that I do not wear as much make-up as I do when I was younger. I use only powder on my face now, nothing more. I just didn’t feel the need for all the makeup anymore (laughs). Though men still complement my looks when I try to look good

INTERVIEWER: How has your husband responded to these changes?

PARTICIPANT 12: Laughs. I really don’t know. My husband in recent times also has his own issues because the natural occurrences is affecting him already. He himself would be 60 in a few months, he is not getting and because of the challenges he has faced in this life in recent times; he aspired for a political post and was disappointed despite sinking all his resources into the campaign, he became frustrated and depressed. He is not showing interest or paying attention to things around him. I am not sure he notices whether I am attractive or not.

**TRANSCRIPT OF PARTICIPANT 13**

**INTERVIEWER:** Good Morning Madam. I am happy that you have consented to participate in this research, and I appreciate your commitment. The first section is meant to help us know each other. Please tell me your name, age, marital status, and your highest level of education.

**PARTICIPANT 13:** I am 53 years old, and married. My highest level of education is a First degree.

**INTERVIEWER:** Thank you. Please note that I will not use your real name in the report, but a coded form. Now, the next section contains questions dealing with the main research. Please let me know if you find it uncomfortable answering any of the questions, so that we may determine how to proceed.

The first area we are going to talk about is about the physical changes have you experienced in your body since you turned 50 years? “How do these changes make you feel”. “How have you expressed these changes to others, including your partner, male colleagues and younger females?” “How do you perceive yourself since you turned 50?”” Do you feel attractive to others?” “Are you attracted to other men?” “What are you doing to remain attractive and cover up your ageing looks?

**INTERVIEWER:** I will start by asking about changes in your body. Have you noticed any change in your body since you turned 50?

**PARTICIPANT 13:** My face is not as fresh as before, I can see some areas of wrinkles already, but I am using a certain cream to smoothen it and make it glow. Also, I look darker in complexion, I am not as light as I used to be, but I know it’s still aging process plus the constant exposure to harsh sun in our climate, so it is to be expected.

Also, I feel pain in my vagina during sexual intercourse which was not so before. I think the pain was caused by dryness in my vagina which I can associate with the onset of menopause which set in 2 years ago when I was 51 years old, because I wasn’t having such pains before. A friend advised that I apply lubricant in my vagina before sexual intercourse, but I haven’t tried it.

**INTERVIEWER:** I suggest you try the lubricant. Because pain will mask the enjoyment and pleasure of sexual intercourse. Is there any other change?

**PARTICIPANT 13:** I sweat a lot much more than before, it comes in form of heat, in which case I now mind the type of clothe that I wear. I wear cotton cloth more than nylon, wool or black cloth or underwear. I keep my windows open; my fan is always working.

**INTERVIEWER:** How do these changes make you feel?

**PARTICIPANT 13:** No, it doesn’t bother me, it doesn’t have any effect on me. I believe the sweating and heat are normal manifestations of menopause. Except at times, when it becomes so unbearable, like clothe becoming so wet while at work. Otherwise, it is reducing in intensity.

**INTERVIEWER:** What about your general attractiveness, do you feel less attractive

**PARTICIPANT 13:** No, I am not less attractive. When you saw me yesterday, you didn’t even believe that I was up to 50. I may not be as pretty as I used to be as I was in my twenties or thirties, but I am still attractive for a woman my age.

**TRANSCRIPT OF PARTICIPANT 14**

**INTERVIEWER:** Good Morning Madam. I am happy that you have consented to participate in this research, and I appreciate your commitment. The first section is meant to help us know each other. Please tell me your name, age, marital status, and your highest level of education.

**PARTICIPANT 14:** I am 54 years old, and married. My highest level of education is a First degree.

**INTERVIEWER:** Thank you. Please note that I will not use your real name in the report, but a coded form. Now, the next section contains questions dealing with the main research. Please let me know if you find it uncomfortable answering any of the questions, so that we may determine how to proceed.

The first area we are going to talk about is about the physical changes have you experienced in your body since you turned 50 years? “How do these changes make you feel”. “How have you expressed these changes to others, including your partner, male colleagues and younger females?” “How do you perceive yourself since you turned 50?”” Do you feel attractive to others?” “Are you attracted to other men?” “What are you doing to remain attractive and cover up your ageing looks?

**PARTICIPANT 14:** The most visible changes that I recognized are in my hair. My hair has become thin, sparse and there are appearances of grey.

**INTERVIEWER:** How do you feel about that?

**PARTICIPANT 14:** It was surprising, because my hair used to be full, long and dark. I could no longer style my hair like I used to do, now I have to be creative in the style or sometimes I wear wig. I am beginning to think of cutting it low. That way I won’t have to worry about it anymore. Thank God, low-cut for ladies is back in vogue. But apart from my hair, I think I am still pretty, if not prettier even (giggles)

**INTERVIEWER:** What can you attribute to this change?

**PARTICIPANT 14:** I can say now that I am over 50, the children are grown, they demand less attention, they can take care of themselves to a large extent and they are mostly away in school, so I have more time for myself. Also, my financial situation is better, the family income has increased so I spend more on myself, I consciously watch my weight so that I don’t put on excess weight. Most importantly, I have peace of mind. The family is settled, and most of the things that I worried about are settled e.g. I used to worry so much over the children. Now, my children are doing fine, the first 2 are gainfully employed and married. The younger 2 are both in higher institutions and I am generally contented with my lot in life.

**INTERVIEWER:** What is your experience with menopause?

**PARTICIPANT 14:** I reached menopause at exactly 50 years. In fact, I didn’t know it was menopause that set in. I got very sick, I had problems with my legs, I couldn’t walk, I thought it was arthritis. I also had serious memory lapse; I forget things easily. It was at the hospital that I was told it was menopausal symptoms. I also had joint pains and back pains. I visited the hospital, I was advised to eat more fruits, exercise more and I was given some drugs which I can’t remember the name now. The problem subsided gradually and it eventually disappeared. Apart from that one, I really did not experience any other problem associated with menopause

**PARTICIPANT 14:** Soon after the menopausal symptoms disappeared, I began to experience an increased urge for sex. I have read it in a magazine that some women may experience such. That was my experience. I think because of the freedom from fear of pregnancy, I felt at liberty to freely have sex. It was a new experience for me because prior to that time, I wasn’t really into it, you know, I was not the bold type that would normally initiate sex, or let my husband know that I want to have sex with him. But after the complete cessation of my menses, I noticed that I was having the urge for sex much more frequently than before. Initially, I was shy and reluctant to let my husband know how I was feeling. I was even embarrassed at first, but later I started expressing my feelings and desire for more sex.

**INTERVIEWER:** I realize these are intimate matters, and I am happy that you are able to freely discuss them with me. But be assured that everything that you say to me shall remain confidential, and will only be used for research purposes alone

**TRANSCRIPT OF PARTICIPANT 15**

**INTERVIEWER:** Good Morning Madam. I am happy that you have consented to participate in this research, and I appreciate your commitment. The first section is meant to help us know each other. Please tell me your name, age, marital status, and your highest level of education.

**PARTICIPANT 15:** I am 56 years old, and separated. My highest level of education is a First degree.

**INTERVIEWER:** Thank you. Please note that I will not use your real name in the report, but a coded form. Now, the next section contains questions dealing with the main research. Please let me know if you find it uncomfortable answering any of the questions, so that we may determine how to proceed.

The first area we are going to talk about is about the physical changes have you experienced in your body since you turned 50 years? “How do these changes make you feel”. “How have you expressed these changes to others, including your partner, male colleagues and younger females?” “How do you perceive yourself since you turned 50?”” Do you feel attractive to others?” “Are you attracted to other men?” “What are you doing to remain attractive and cover up your ageing looks?

**INTERVIEWER:** Since you turned 50, have you experienced any changes in your body?

**PARTICIPANT 15:** Not much, when I’m sleeping, I always feel pains in my bones, especially in my legs, my bone will be paining me in the night, I wouldn’t want to stand up or I feel reluctant to stand up from sleep. I always have bone whatever; I don’t know how to put it

**INTERVIEWER:** What other changes have you observed?

**PARTICIPANT 15:** Hmm, you know I have not been menstruating again. Actually, the menstruation stopped since when I was 49 years. Following menopause, I started having hot flashes, I was sweating excessively and I experienced some memory problems. I started forgetting things a lot.

**INTERVIEWER:** How did that make you feel?

**PARTICIPANT 15:** Initially, I was scared; I would leave the kitchen to go and take something from the bedroom, and in the middle of the way, I wouldn’t remember what I was going to do, or I would summon a junior teacher to my office, and when he arrives, I wouldn’t remember why I invited him. It was worrisome, and sometimes embarrassing. I stopped worrying about it when I realized it is a normal process of aging and that it sometimes follows menopause. I then devise a method of coping at work, I would jot things down as it comes to my mind.

**INTERVIEWER:** Have you noticed further changes regarding menstruation since you turned 50?

**PARTICIPANT 15:** The complaints continued past age 50. Though in the last 2 years or so, I have fewer complaints, the hot flashes and sweating has reduced, I only experience that occasionally now, or maybe I have gotten used to the feeling. I still forget things though (laughs).

**INTERVIEWER:** Are there any more changes that you observe in your body since you turned 50?

**PARTICIPANT 15:** Not really. I am happy with my body and my looks. I became conscious of my looks when people started remarking how youthful I look. I started paying more attention to my body and appearance because I love the complements I get from people regarding my look. I would say that I love the way I look. I believe the society, especially, men and the youth appreciate women who are able to remain youthful, slim, without wrinkles, and grey-hairs, but also, they are quick to remind you to “act your age.” if you step out of line.

**TRANSCRIPT OF PARTICIPANT 16**

**INTERVIEWER:** Good Morning Madam. I am happy that you have consented to participate in this research, and I appreciate your commitment. The first section is meant to help us know each other. Please tell me your name, age, marital status, and your highest level of education.

**PARTICIPANT 16:** I am 58 years old, and widowed. My highest level of education is a First degree.

**INTERVIEWER:** Thank you. Please note that I will not use your real name in the report, but a coded form. Now, the next section contains questions dealing with the main research. Please let me know if you find it uncomfortable answering any of the questions, so that we may determine how to proceed.

The first area we are going to talk about is about the physical changes have you experienced in your body since you turned 50 years? “How do these changes make you feel”. “How have you expressed these changes to others, including your partner, male colleagues and younger females?” “How do you perceive yourself since you turned 50?”” Do you feel attractive to others?” “Are you attracted to other men?” “What are you doing to remain attractive and cover up your ageing looks?

**INTERVIEWER:** Have you noticed any changes in your body since you turned 50?

**PARTICIPANT 16:** Yes, quite a few changes. The most remarkable is the fact that I don’t have much strength like I did when I was younger. I get tired with little exertion. When I was 50 years, I had a fibroid operation, which involved complete removal of my reproductive organs. Thereafter, I got into menopause unprepared. Soon after, I started experiencing sudden body heat, sweating, vagina dryness, fluctuation of mood and sometimes memory issues.

**INTERVIEWER:** How did you feel about these changes?

**PARTICIPANT 16:** I realized that those changes came as a result of hormonal changes which was a result of the sudden onset of menopause, and that it will fade away gradually. So, I bore it while it lasted. All the symptoms have disappeared except for the memory and forgetfulness. Though, I wouldn’t know about the dry vagina since I haven’t been sexually active since my husband died 4 years ago.

**INTERVIEWER:** What about the way you look? Is there any change in the way you look?

**PARTICIPANT 16:** I have gained weight since I turned 50. Especially in the year following my husband’s death. I completely let go. I lost interest in my look. I stopped dressing up and I started eating more than necessary. I realized I had become very fat and unattractive, and my blood sugar level became higher than normal. My blood pressure was also high for my age. I tried to correct that. I started to pay attention to my looks, and for health reasons, I had to work on the extra weight.

**INTERVIEWER:** What did you do about these conditions?

**PARTICIPANT 16:** I started working on shedding the extra weight and controlling my blood sugar level by watching what I eat and taking 30 minutes’ walk every morning. I need to look good for myself and health reasons. I also had to go on a special diet and exercise programme for weight reduction. Now, my weight and my blood pressure are all fine.

**INTERVIEWER:** Are you satisfied with your weight now?

**PARTICIPANT 16:** Yes. I’m satisfied, you know that I don’t have husband now, what else am I going to use all that trim figure for? I have no one to impress. But I’m okay

**INTERVIEWER:** Any other change, like in your look; In your complexion? Are there wrinkles? Is your hair growing thinner or fuller?

**PARTICIPANT 16:** My hair isn’t as full as before. Though I still have some hair. I haven’t completely gone bald. This is all my hair; I have no attachments on.

**INTERVIEWER:** Are okay with this?

**PARTICIPANT 16:** Yes. I am quite happy with my hair. I can still make my hair into different styles. I am particularly happy because the hair is still dark. Grey hair somehow adds to your age. (laughs). I plan to cut my hair and wear a low cut when I turn 60.

**TRANSCRIPT OF PARTICIPANT 17**

**INTERVIEWER:** Good Morning Madam. I am happy that you have consented to participate in this research, and I appreciate your commitment. The first section is meant to help us know each other. Please tell me your name, age, marital status, and your highest level of education.

**PARTICIPANT 17:** I am 57 years old, and married. My highest level of education is a First degree.

**INTERVIEWER:** Thank you. Please note that I will not use your real name in the report, but a coded form. Now, the next section contains questions dealing with the main research. Please let me know if you find it uncomfortable answering any of the questions, so that we may determine how to proceed.

The first area we are going to talk about is about the physical changes have you experienced in your body since you turned 50 years? “How do these changes make you feel”. “How have you expressed these changes to others, including your partner, male colleagues and younger females?” “How do you perceive yourself since you turned 50?”” Do you feel attractive to others?” “Are you attracted to other men?” “What are you doing to remain attractive and cover up your ageing looks?

**PARTICIPANT 17:** Since I turned fifty, I have experienced a lot of increase in my weight. I have become much fatter than I used to be. My tummy is also bigger.

**INTERVIEWER:** How do you feel about the changes?

**PARTICIPANT 17:** I feel terrible about the way I look now. I mean, you wake up one day, you look at yourself in the mirror and you see this creature you almost didn’t recognize. She has double chin, loose, sagging skin around the face, sagging breasts, extra fat around the middle that wasn't there before. Skin turns dry, cellulite spreading all over, veins popping up on the legs and worse of all, there’s difficulty remembering simple things. But it’s strange how one doesn’t feel old in one’s mind. In my mind, I feel no differently from before, but when I look in the mirror, I see an older woman, but I’m working on getting my old, trimmer physique back.

**INTERVIEWER:** I am sorry you feel terrible about these changes. What do you want to do about them?

**PARTICIPANT 17:** I realized that I continued to gain weight even though I wasn’t eating much, and despite that I do exercise, though irregularly. I later realized that my metabolism has really slowed down following menopause. Because the extra fat around my waist and abdomen makes me look older than I am, I now engage in abdominal and waist exercises to get my figure back and renew my youthful look. This is helping. My abdomen is reducing slowly.

I also have pains in my legs. I understand it might be arthritis because of my heavy weight. I also noticed my hair is breaking, its scantier, less full and thick like before. I also see the appearance of grey hair.

**INTERVIEWER:** What are you doing about all these changes?

**PARTICIPANT 17:** I have been advised to watch my weight by reducing my intake of starch foods and by exercising regularly. Though, these are not easy measures, but I’ll try.

**INTERVIEWER:** The appearance of grey hair, how does that make you feel?

**PARTICIPANT 17:** I’m happy about it, that at least I’m getting old, I am a matured adult, something like that. I have lots of experiences to show for it, that cannot be bought with money I think I have earned all my scars. I am happy

**INTERVIEWER:** When did you enter into menopause?

**PARTICIPANT 17:** About 5 years ago

**INTERVIEWER:** What other changes did you see after the menopause?

**PARTICIPANT 17:** I find it more difficult to get a good night sleep because of menopausal symptoms like hot flashes and night sweats.

**INTERVIEWER:** What are you doing about that?

**PARTICIPANT 17:** I mind the material of the clothe I wear and I take my portable, rechargeable hand fan with me everywhere I go. Though the heat has reduced greatly.
