# Supplementary material for: An Interpretative Phenomenological Analysis of the lived experience of sensuality expression among women over 50 years of age in Nigeria
Source: PLoS One. 2023 Jun 1;18(6):e0285362. doi: 10.1371/journal.pone.0285362 (PMC10234539; doi:10.1371/journal.pone.0285362)
Supplement: Attachment — Submitted filename: RESPONSE TO REVIEWERS.docx [file pone.0285362.s002.docx]

| RESPONSE TO REVIEWERS | | |
| --- | --- | --- |
| **REVIEWER 1** | | |
| NO | QUERRY | RESPONSE |
| 1. | While the authors conducted a pilot study, I would strongly suggest not to include the pilot study data into the analysis of the study. Perhaps the authors might want to explain the choice of doing that. | The pilot data were included in the analysis of the main study as the instrument did not change substantially, and there is no potential risk of contamination that could compromise the quality of the findings [25]. This is allowed in qualitative studies where there is no risk of data contamination. (Lines 190-194).  The potential risk identified was possible reluctance to disclose sensitive matters during face-to-face interviews. This was prevented in the main study by assuring participants of confidentiality, and conducting the interviews in safe and supportive environment of the participant’s choice Lines (179 – 182) |
| 2. | Judgment sampling often does have a possible bias. This may need to be noted in the limitations of the study. | To avoid possible bias, the participants were encouraged to freely give their consent to participate in the study, following adequate information about their expectations. (Line 669 – 671) |
| 3. | Since sensuality can be a taboo subject, we may never know for certain if the participants were being truthful in the interviews. Could stigma around the topic sway the participants’ responses? | Stigma, defined by Oxford Dictionary as “a mark of disgrace associated with a particular circumstance’’ may not be applicable to this phenomenon. Sensuality; an essential component of sexuality, is an integral part of being human. Though it has been established that society may not readily accept the expression of sexuality by women over 50 years, and that the women themselves oftentimes accept this stereotype; but it is not documented that they feel disgraced or stigmatized as a result of expressing their sensuality. Also, in reference to the structured interview questions asked, t**here were no perceived stigma attached to these questions (Line 201-209).** |
| 4. | The authors may need to proof edit the document. | Done |
| 5. | I suggest the authors provide a citation to accompany the definition of sensuality. | Done (Line 50) |
| 6. | Given the work that the authors have done in this manuscript it may help if they could make more specific recommendations, addressing how this research might help nurses research or how this research helps to fill the research gap. What other research might need to be done to close the gap?” | In order to generate more knowledge in this area of study, we suggest a larger study using quantitative methods so as to reach a larger population of women who would relate their lived experiences of this phenomenon. Further qualitative studies may be undertaken to explore other aspects of sexuality of women in this age bracket for the development of deeper and comprehensive nursing framework to address their sensuality.  Furthermore, this study covered one state out of the six in the Southwest Geopolitical zone of Nigeria. Replicating this study in the other states in the zone will be more representative of women of that culture (Line 675 – 683).  By using their voices, the researchers have broken the silence about the sensuality needs of this group and their perceived invisibility in the eyes of the society (Line 664– 666). |
| **REVIEWER 2** | | |
| 1. | Introduction and Study Focus - The authors need to expand on the relevance of this topic and build up a stronger justification for researching this. | It is well documented that the subject of sexuality of women above 50 is often overlooked, neglected, and unspoken [3, 4], despite this being a lifelong expression that plays an important role in graceful ageing due to its physiological, psychological and emotional benefits, its contribution to a person’s dignity and social life, and a direct link to increased quality of life [5] (Lines 63 – 69).  It is also documented that the stereotype of the sexless older person has continued to influence policy making and research agendas [16] (Lines 96 – 100).  This study projects the voice of this group of women as they relay their lived experiences on the expression of their sensuality, thereby providing knowledge and breaking the silence about the peculiar sensuality needs of this group of women, and their perceived invisibility in the society (Lines 111-114). |
| 7. | It is unclear to me the applied value that the researchers aim for. In the abstract, there is a reference to “… the development of age-appropriate healthy-ageing interventions,” but the study is exploratory, and at best, can generate questions for further studies to understand the topic. In addition, in order to hint at the development of “… further sexual health intervention research,” the authors could have included a subgroup of women with sexuality problems in their study. | This is not an intervention study. The main focus of this study is to project the voice of this group of women on the expression of their sensuality, thereby breaking the silence about the peculiar sensuality needs of this group of women, and their perceived invisibility in the society.  Larger studies may be undertaken to explore other aspects of sexuality of women in this age bracket for the development of deeper and comprehensive nursing framework to address their sensuality (Lines 675 – 683). |
| 8. | Research Design - This part needs more detail. The IPA methodology for data collection requires further explanation | Further explanation on IPA as a methodology done (Lines 126 – 144). |
| 9. | The manuscript needs editing | Done |
| 10. | The Yoruba people in the south western part of Nigeria is an ethnic group with sub-dialects, NOT a sub-tribe. | Corrected (Line 212) |
| 11. | Reference in upper case | Corrected (Lines 733 -734, 809-810) |
